# Open Mesh Repair of a Primary Grynfelt-Lesshaft Hernia: A Case Report and Literature Review

**DOI:** 10.7759/cureus.113159

**Published:** 2026-07-22

**Authors:** Diego Andres De León Rosas, Mariana Treviño Ayala, Bernardo Alfonso Fernández-Reyes, Raul R Leal Mier, Ramiro Gonzalez Lozano

**Affiliations:** 1 General Surgery, Hospital Christus Muguerza Alta Especialidad, Monterrey, MEX

**Keywords:** abdominal wall defect, grynfelt–lesshaft hernia, lumbar hernia, mesh repair, superior lumbar hernia

## Abstract

Grynfelt-Lesshaft hernias are extremely rare posterolateral abdominal wall defects, with a nonspecific clinical presentation that often leads to misdiagnosis or delayed recognition; hence, a high index of suspicion and proper imaging are necessary.

A 71-year-old woman presented with a gradually increasing swelling over the left lumbar region for three months. On examination, there was a soft, reducible, nontender swelling in the left posterolateral lumbar region, which increased after performing Valsalva's maneuver. Ultrasound of the abdomen revealed a 4 cm defect in the left superior lumbar triangle with protruding retroperitoneal fat. A non-contrast-enhanced CT scan confirmed the presence of a primary Grynfelt-Lesshaft hernia containing retroperitoneal fat without any visceral involvement or incarceration. Open elective repair was performed via an 8 cm left subcostal incision with the patient positioned on their right side. The hernia sac was dissected, the herniated contents were reduced, and the defect was repaired with primary suture and a polypropylene mesh that was placed in a subaponeurotic plane and secured to the periosteum of the 12th rib, transversalis fascia, quadratus lumborum muscle, and internal oblique muscle. The patient had an uneventful postoperative course with adequate pain control and no wound-related complications. Clinical recovery was satisfactory at the one-year follow-up.

Grynfelt-Lesshaft hernia is an entity that poses a challenge for diagnosis because of its rarity and variable clinical presentation. Computed tomography has remained the diagnostic modality of choice. It helps confirm the diagnosis of hernia, define its contents, and aid in planning the surgery. This case supports open tension-free mesh repair as a feasible option in appropriately selected primary superior lumbar hernias.

## Introduction

Lumbar hernias are rare abdominal wall posterolateral defects, constituting less than 2% of all abdominal wall hernias. They occur through areas of anatomic weakness in the lumbar region, most commonly the superior lumbar triangle of Grynfelt-Lesshaft and, less commonly, the inferior lumbar triangle of Petit. Due to its rarity, most surgeons encounter only a limited number of lumbar hernias during their careers, contributing to delayed diagnosis and the absence of standardized management strategies [1,2].

The superior lumbar hernia is the most common type of primary lumbar hernia. Weakness in this region allows for the protrusion of retroperitoneal fat, colon, kidney, or small bowel through the defect in the fascia. Patients usually provide a history of a slow enlargement of a swelling in the lumbar region, which is more apparent during physical exercise or Valsalva's maneuver and may be associated with discomfort, backache, or cosmetic concerns [2].

Because of their nonspecific clinical presentation, lumbar hernias are frequently misdiagnosed as a lipoma, soft tissue tumor, hematoma, abscess, or other lumbar mass. Recent reports continue to underscore the diagnostic challenges that this condition poses, underlining the need for a high index of suspicion. At present, computed tomography has assumed the role of the imaging modality of choice because it accurately delineates the fascial defect, characterizes the contents of the hernia, evaluates the neighboring structures, and helps in preoperative planning. Ultrasonography may be a good initial diagnostic test; however, cross-sectional imaging is considered mandatory for the final diagnosis and surgical decision-making [3,4].

To date, surgical repair is considered the definitive treatment. It is generally recommended even in patients with minimal symptoms because of the risk of progressive enlargement, incarceration, and strangulation [2]. Despite descriptions of several surgical techniques, which include open, laparoscopic, retroperitoneoscopic, and robotic approaches, the optimal management strategy has not yet been established due to the limited number of reported cases [1,2]. Open tension-free mesh repair remains a safe, effective, and reproducible option, particularly for small- and moderate-sized defects, with favorable outcomes and low recurrence rates reported in recent series [1,5].

We discuss the management of an elderly patient with a primary left superior lumbar hernia through the Grynfelt-Lesshaft triangle, successfully treated by open mesh repair. We also discuss the diagnostic and therapeutic considerations due to the rarity of primary superior lumbar hernias and provide a diagnostic pathway, from clinical suspicion to ultrasound and confirmatory CT, culminating in elective repair with detailed technical points of mesh fixation. 

## Case presentation

A 71-year-old woman with a history of long-standing hypothyroidism under medical treatment presented for evaluation of a progressively enlarging mass in the left costolumbar region. Her body mass index (BMI) was 28.7 kg/m², consistent with overweight. She denied recent weight loss and had no history of chronic cough, chronic obstructive pulmonary disease, constipation, excessive straining, heavy lifting, smoking, abdominal or flank surgery, or prior trauma. The condition began approximately three months before presentation and was characterized by the insidious onset of a left lumbar mass that progressively increased in size with maneuvers that elevated intra-abdominal pressure, particularly during the Valsalva maneuver, and partially decreased at rest. The patient denied local pain, skin discoloration, asthenia, adynamia, irritative urinary symptoms, or changes in bowel habits.

Upon admission, the patient was hemodynamically stable. Physical examination revealed a soft, reducible, non-tender mass measuring approximately 4 × 4 cm in the left lumbar region without overlying skin changes. The bulge became more prominent during the Valsalva maneuver and in the standing position and decreased with manual reduction and in the supine position.

**Figure 1:**
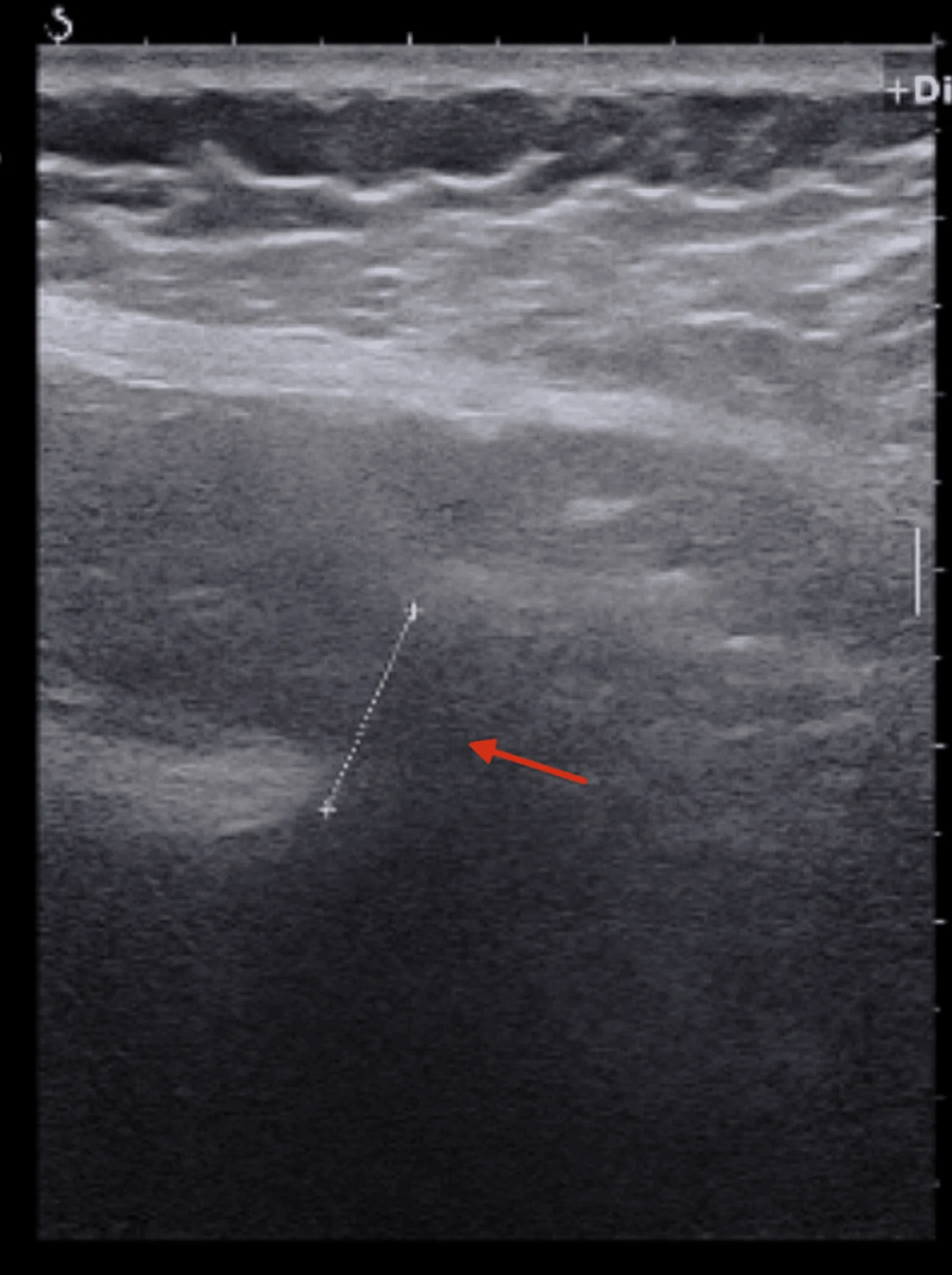
Abdominal ultrasound A 4-cm defect within the left superior lumbar triangle (arrow), associated with protrusion of fatty tissue through the abdominal wall, forming a hernia sac measuring approximately 7 × 2 cm.

**Figure 2:**
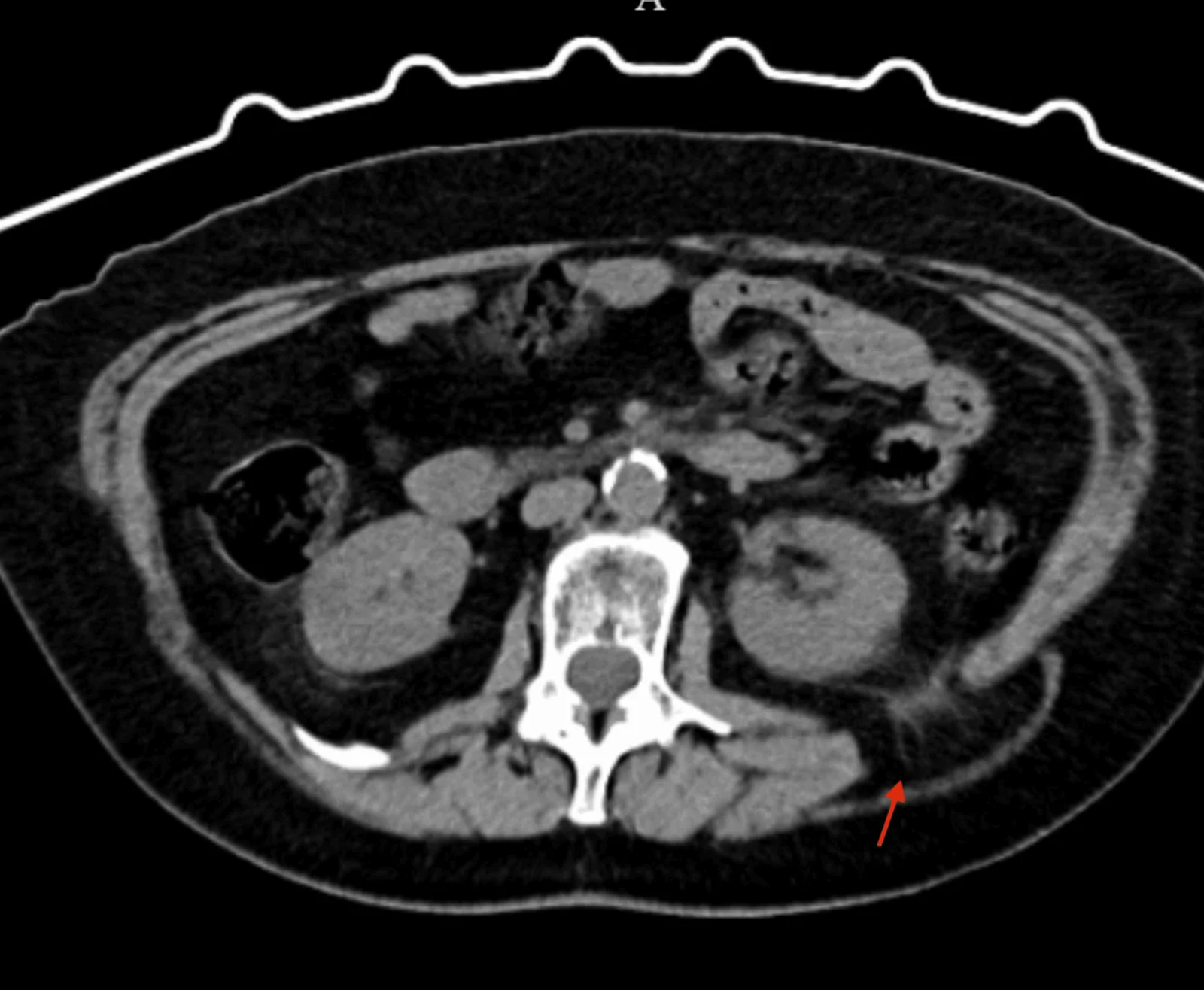
Non-contrast abdominal CT, axial view Superior lumbar hernia defect (arrow) containing retroperitoneal fat without evidence of visceral involvement or incarceration.

**Figure 3:**
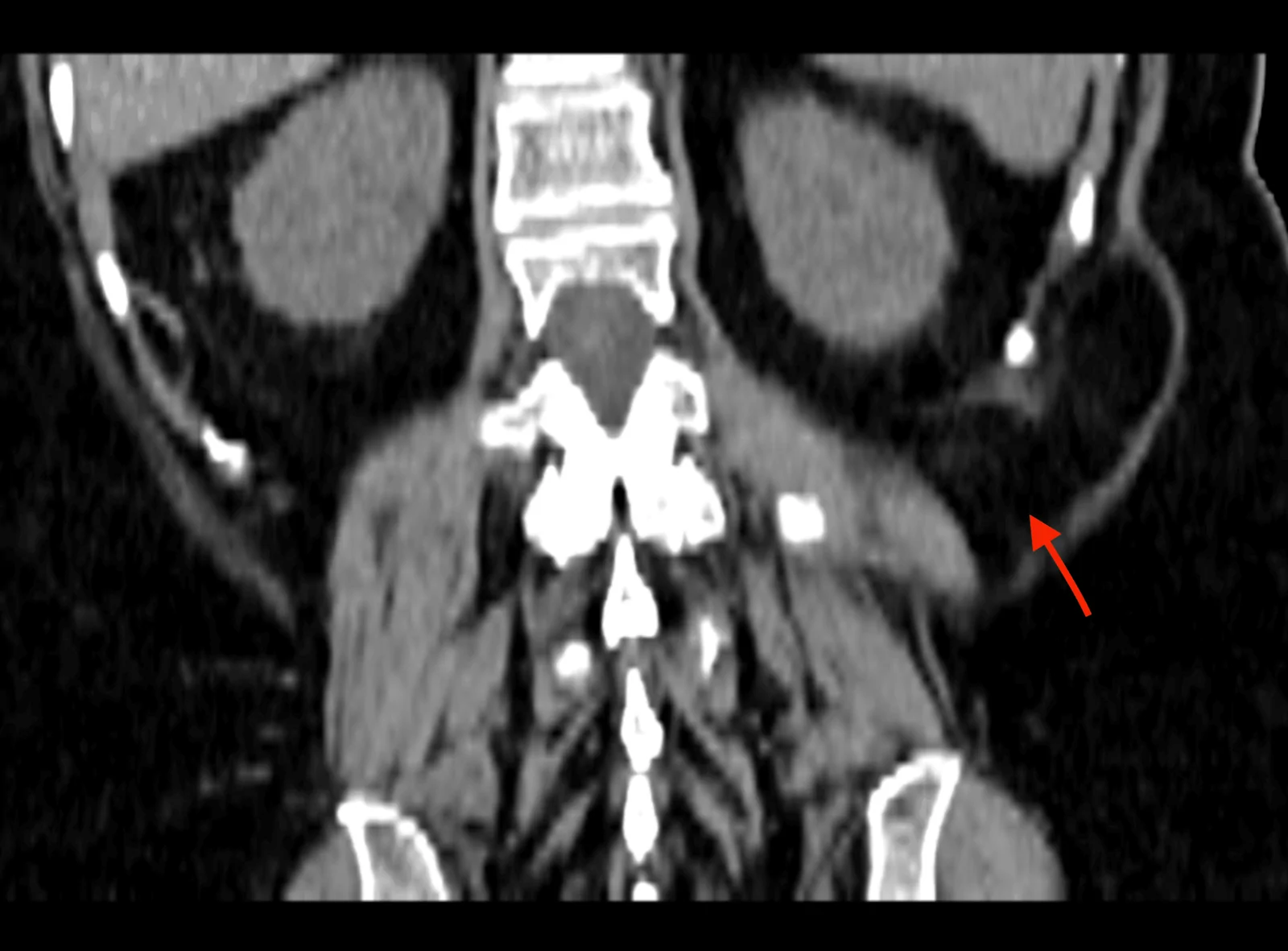
Non-contrast abdominal CT, coronal reformation Superior lumbar triangle defect (arrow) with retroperitoneal fat herniation, without visceral involvement or signs of incarceration.

Preoperative laboratory investigations, including complete blood count, serum chemistry profile, liver function tests, and coagulation studies, were within normal limits. An abdominal ultrasound performed in July 2025 demonstrated an approximately 4-cm defect within the left superior lumbar triangle, associated with protrusion of fatty tissue through the abdominal wall, forming a hernia sac measuring approximately 7 × 2 cm. These findings were further evaluated with non-contrast-enhanced abdominal computed tomography, which confirmed the presence of a superior lumbar (Grynfelt-Lesshaft) hernia containing retroperitoneal fat without evidence of visceral involvement or incarceration. Based on the clinical and imaging findings, a diagnosis of primary left superior lumbar hernia was established (Figures 1-3).

Elective open repair was performed under general anesthesia with the patient in the right lateral decubitus position. Prophylactic antibiotic therapy was administered with 2 g of intravenous cefalotin prior to skin incision. Through an 8-cm left subcostal incision below the 12th rib, dissection was carried down to the muscular plane, revealing a 7 × 4 cm hernia sac within the superior lumbar triangle. Careful dissection allowed identification of the anatomical boundaries of the Grynfelt-Lesshaft triangle, including the 12th rib superiorly, the quadratus lumborum muscle medially, and the posterior border of the internal oblique muscle laterally. The floor of the defect was formed by the transversalis fascia, while the latissimus dorsi muscle constituted the superficial boundary. The hernia contents consisted of preperitoneal fat, which was reduced without difficulty.

**Figure 4:**
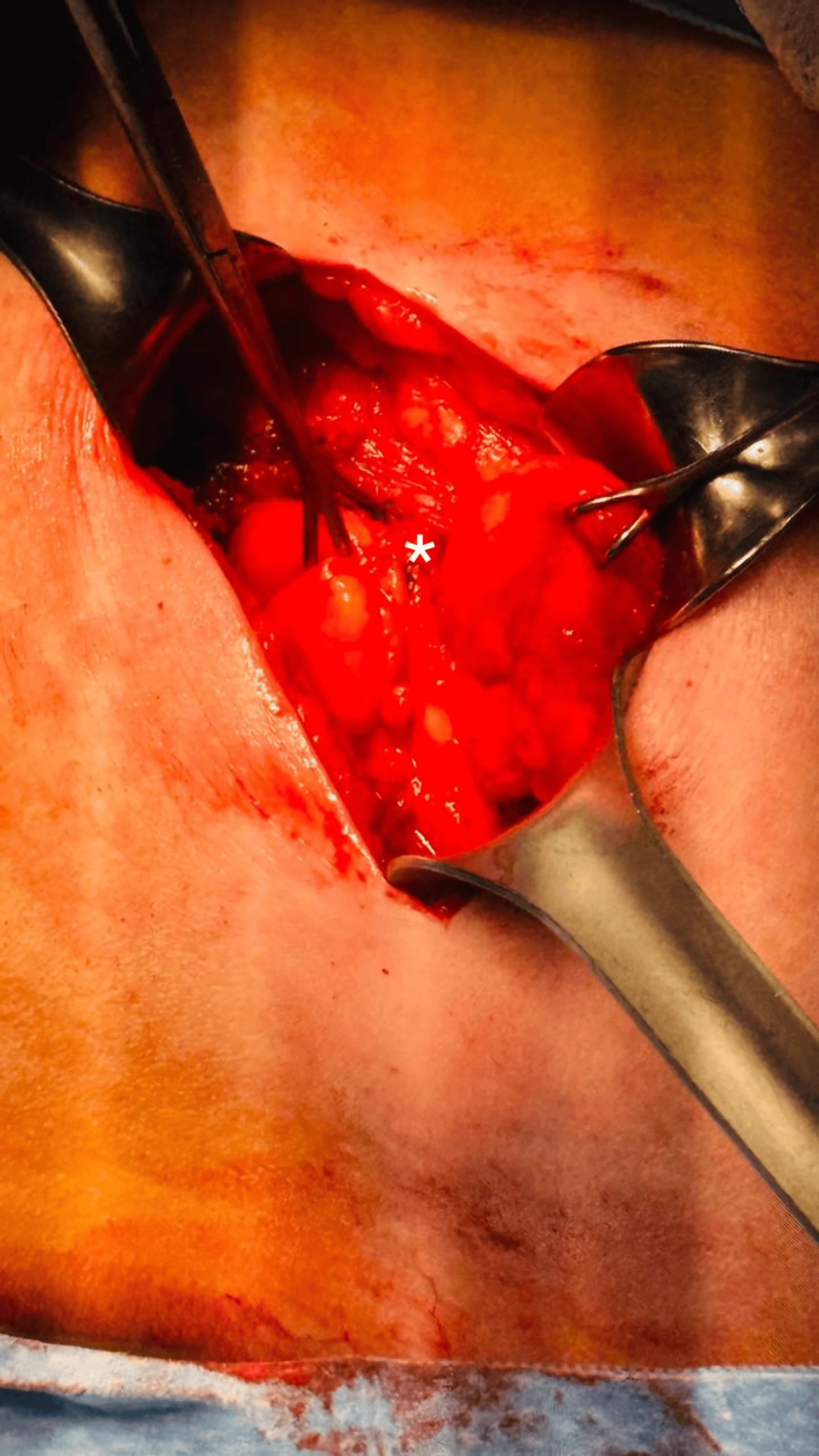
Surgical view of hernia defect Hernial sac and fatty tissue content (*)

**Figure 5:**
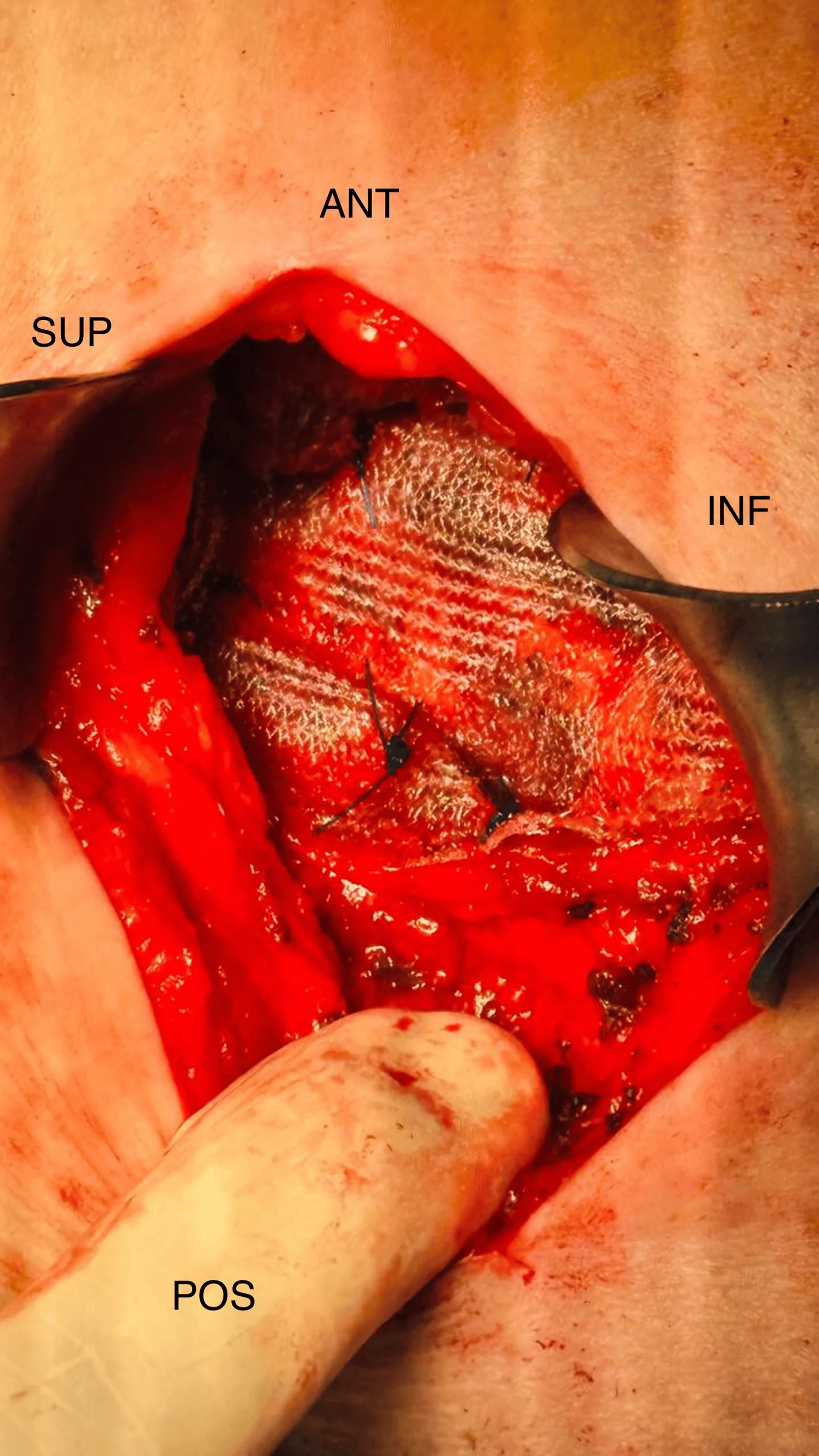
Open repair with prosthetic mesh reinforcement ANT: anterior; SUP: superior; INF: inferior; POS: posterior. Polypropylene sutures were placed at the fixation point, 12th rib, transversalis fascia, quadratus lumborum muscle, and internal oblique muscle.

Primary closure of the defect was performed using a continuous 2-0 polyglactin (Vicryl®) suture. Reinforcement was achieved with a 7 × 7 cm polypropylene mesh (Promech®) that was tailored and placed in a subaponeurotic position with a 3 cm defect overlap. The mesh was secured to the periosteum of the 12th rib, transversalis fascia, quadratus lumborum muscle, and internal oblique muscle using 2-0 polypropylene (Prolene ®) sutures, providing adequate coverage of the defect. After confirmation of hemostasis, layered closure was completed using 2-0 Vicryl® for the fascia and latissimus dorsi muscle, 2-0 and 3-0 Vicryl® for the subcutaneous tissue, and a 4-0 intradermal Vicryl® suture for skin closure; no surgical drains were used (Figures 4-5). The procedure was completed without intraoperative complications, with an estimated blood loss of 15 ml. The patient had an uneventful postoperative course and was discharged on postoperative day 2. After a year of follow-up, no chronic pain, surgery-related complications, or hernia recurrence were observed.

## Discussion

Since the first description of lumbar hernias by Garangeot in 1731, few cases have been reported worldwide. This explains why the majority of general surgeons have limited clinical exposure, and there are no universally accepted therapeutic guidelines [1,2]. This case demonstrates the diagnostic challenge due to the rarity of primary superior lumbar hernias and provides a diagnostic pathway, from clinical suspicion to ultrasound and confirmatory CT, culminating in elective repair with detailed technical points of mesh fixation to the 12th rib periosteum, transversalis fascia, quadratus lumborum, and internal oblique muscle.

Lumbar hernias are anatomically classified according to the lumbar triangle through which they protrude. The inferior lumbar triangle (Petit’s triangle) is bounded by the latissimus dorsi muscle posteriorly, the external oblique muscle anteriorly, and the iliac crest inferiorly. In contrast, the superior lumbar triangle, also known as the Grynfelt-Lesshaft triangle, is bordered superiorly by the 12th rib, medially by the quadratus lumborum and erector spinae muscles, and laterally by the internal oblique muscle. Its floor is formed by the transversalis fascia and the aponeurosis of the transversus abdominis muscle, whereas its roof is composed primarily of the latissimus dorsi muscle [2,6]. Although inferior lumbar hernias were historically considered more common, studies have demonstrated that Grynfelt-Lesshaft hernias represent the predominant subtype. This higher prevalence has been attributed to several anatomical weak points within the superior lumbar triangle, including the area immediately below the 12th rib, the passage of the subcostal neurovascular bundle, and the space between the inferior border of the 12th rib and Henle’s ligament [2,6].

About 20% of lumbar hernias are considered to be congenital, developing from embryological anomalies. The acquired variety is further classified into primary and secondary. Secondary lumbar hernias are often due to trauma, previous surgery, infections, or muscular defects following surgical incisions. Predisposing factors are advanced age, obesity, marked weight loss, muscular atrophy, chronic debilitating diseases, chronic bronchitis, postoperative sepsis, and constant rise in intra-abdominal pressure [1,2,7]. The age of our patient is consistent with the demographic profile most commonly reported in the literature. Likewise, the left-sided location observed in this case is in agreement with previous reports describing a predominance of unilateral left-sided lumbar hernias [2,4]. No history of trauma or surgery in the lumbar region supports the diagnosis of primary acquired lumbar hernia.

The clinical presentation of lumbar hernias is often nonspecific. Most patients present with a slowly enlarging lumbar mass that becomes more prominent during the Valsalva maneuver and decreases at rest. Some patients complain of lumbar discomfort or pain, or they feel a sensation of heaviness over the lumbar area. Other patients may remain asymptomatic for a long period. Because of this variable presentation, the differential diagnosis includes lipomas, hematomas, abscesses, fibromas, sarcomas, pseudohernias secondary to muscular denervation, and other retroperitoneal masses [2,4,6]. Clinical diagnosis of lumbar hernias may be difficult, especially in obese individuals and in patients with small or occult defects. Therefore, imaging studies are often required, and computed tomography scans are considered the gold standard for diagnosis because they have high sensitivity and can accurately delineate the fascial defect as well as the contents of the hernia [2,4].

Computed tomography is considered the gold standard for diagnosing lumbar hernias. Its diagnostic value lies in its ability to demonstrate the fascial defect, define hernia contents, evaluate adjacent structures, and guide operative planning. In addition, CT imaging facilitates the identification of potential complications, including incarceration and strangulation. Ultrasonography is a readily available and low-cost imaging study that may be used as the primary modality, especially when the hernia contains predominantly retroperitoneal fat [3,4].

Once the diagnosis is established, surgical repair is generally recommended because of the risk of progressive enlargement, incarceration, and strangulation. Reported incarceration risk may reach 25%, whereas strangulation has been described in 8-18% of cases, depending on the size of the defect and hernia contents [2,7,8]. Although most patients do not initially present with acute complications, the potential for progression supports early operative management. Moreno-Egea et al. proposed a widely accepted preoperative classification system for lumbar hernias, incorporating defect size, anatomical location, hernia contents, etiology, degree of muscular atrophy, and recurrence status. Beyond providing a standardized description, this classification has therapeutic implications and may assist in selecting the most appropriate surgical approach. Based on its small size, primary etiology, absence of muscular atrophy, lack of recurrence, and herniation limited to retroperitoneal fat, our case was consistent with a Type A lumbar hernia. This subtype is defined by small defect size (<5 cm), superior location, spontaneous etiology, and favorable anatomy, making it particularly suitable for open repair with prosthetic reinforcement [7]. These features further support the surgical strategy selected for our patient.

The optimal surgical approach for lumbar hernia repair has not yet been universally agreed upon. Options include conventional open repair, open mesh repair, sublay techniques, transabdominal laparoscopic repair, totally extraperitoneal approaches, and more recently, robotic-assisted procedures. Systematic reviews and case series have suggested that minimally invasive approaches may be associated with reduced postoperative pain, shorter hospital stays, and earlier recovery [1,2,5,9]. However, the available evidence remains limited due to the rarity of the condition and the lack of large comparative studies.

Presentation in an elderly woman, left-sided localization, and protrusion of retroperitoneal fat as the sole hernia content correspond to the most frequently described findings in the literature [4,10]. Recent reports illustrate the heterogeneous clinical spectrum of Grynfelt-Lesshaft hernias. Mehdaoui et al. described a complicated superior lumbar hernia associated with recurrence, bowel obstruction, colonic necrosis, perforation, and intra-abdominal sepsis, highlighting potentially severe consequences of delayed diagnosis or treatment [8]. In contrast, our patient was diagnosed before the development of incarceration or visceral compromise and underwent elective repair with an uneventful postoperative course. Osman et al. reported a case of recurrent bilateral lumbar hernia and underlined the diagnostic difficulties and rarity of recurrence in this condition [11]. These reports reinforce the importance of early recognition, appropriate imaging, and individualized surgical planning.

Open repair remains a valid and widely utilized option, particularly for small- and medium-sized defects, where excellent exposure and secure prosthetic fixation can be achieved [1,5,7]. In the present case, an open approach was selected because of the anatomical characteristics of the defect and the possibility of obtaining robust fixation to the periosteum of the 12th rib, transversalis fascia, quadratus lumborum muscle, and internal oblique muscle. The use of a polypropylene mesh reinforced the primary repair and potentially reduced the risk of recurrence. At the one-year follow-up, the patient remained asymptomatic, with no evidence of hernia recurrence or postoperative complications. Some recent reports have described successful outcomes with similar techniques. Cabrera et al. reported a young female patient with a Grynfelt-Lesshaft hernia. The condition was treated through an open lumbar approach, and polypropylene mesh was placed; there was no recurrence after six months of follow-up [10]. Similarly, Ganesan et al. reported successful surgical management of an elderly patient with a Grynfelt-Lesshaft hernia. These results further support that open mesh repair is safe and effective in appropriately selected patients [12].

Despite recent advances in laparoscopic and robotic surgery, the rarity of lumbar hernias has limited the development of high-quality comparative studies. Consequently, the choice of surgical approach continues to depend on defect characteristics, surgeon expertise, and institutional resources. In our patient, open repair provided excellent exposure of the lumbar anatomy and allowed secure mesh fixation, resulting in a successful postoperative outcome without recurrence during early follow-up [1,2,9]. This outcome observed in our patient is consistent with recent reports and further supports open mesh repair as a safe, reproducible, and effective treatment option for selected patients with primary Grynfelt-Lesshaft hernias [1,5,10,11]. The decision to perform an open repair was guided by the surgical team’s experience and proficiency with this approach, which was considered the most appropriate strategy for this patient. 

## Conclusions

The superior lumbar hernia of Grynfelt-Lesshaft is an uncommon abdominal wall defect, whose rarity contributes to delayed diagnosis. With a nonspecific clinical presentation, it requires a high index of suspicion and the support of imaging studies. Ultrasonography may serve as a useful initial diagnostic tool; however, computed tomography remains the imaging modality of choice for confirming the diagnosis, defining the hernia contents, identifying potential complications, and facilitating surgical planning. Once the diagnosis is established, surgical repair is generally considered the treatment of choice because of the risk of progressive enlargement and potential complications.
